# *In vivo* Characterization of *Plasmodium berghei* P47 (Pbs47) as a Malaria Transmission-Blocking Vaccine Target

**DOI:** 10.3389/fmicb.2020.01496

**Published:** 2020-07-03

**Authors:** Lampouguin Yenkoidiok-Douti, Gaspar E. Canepa, Ana Beatriz F. Barletta, Carolina Barillas-Mury

**Affiliations:** ^1^Laboratory of Malaria and Vector Research, National Institute of Allergy and Infectious Diseases (NIAID), National Institutes of Health, Rockville, MD, United States; ^2^Fischell Department of Bioengineering, University of Maryland, College Park, MD, United States

**Keywords:** malaria, P47, transmission-blocking vaccines, VLPs, passive immunization, *Anopheles gambiae*, *Plasmodium*

## Abstract

An effective vaccine to reduce malaria transmission is central to control and ultimately achieve disease eradication. Recently, we demonstrated that antibodies targeting the *Plasmodium falciparum* surface protein P47 (Pfs47) reduce parasite transmission to *Anopheles gambiae* mosquitoes. Here, *Plasmodium berghei* (Pb) was used as a model to assess the *in vivo* efficacy of a P47-targeted transmission blocking vaccine (Pbs47). Mice were immunized following a prime/boost regimen and infected with *P. berghei*. The effect of immunization on infectivity to mosquitoes was evaluated by direct feeding on *P. berghei*-infected mice. The key region in Pbs47 where antibody binding confers protection was mapped, and the immunogenicity of this protective antigen was enhanced by conjugation to a virus-like particle. Passive immunization with 100 and 50 μg/mL of anti-Pbs47 IgG reduced oocyst density by 77 and 67%, respectively. Furthermore, affinity purified Pbs47-specific IgG significantly reduced oocyst density by 88 and 77%, respectively at doses as low as 10 and 1 μg/mL. These studies suggest that P47 is a promising transmission blocking target and show that antibodies to the same specific region in Pfs47 and Pbs47 confer protection.

## Introduction

Malaria is an infectious disease caused by *Plasmodium* parasites and transmitted by the bite of infected *Anopheles* mosquitoes (Phillips et al., 2017). The widespread deployment of effective interventions, including antimalarial drugs and insecticides for vector control, over the last two decades led to significant reductions in malaria burden. In 2018, the WHO estimated 228 million cases of malaria worldwide leading to 405,000 deaths, compared with 262 million cases and 839,000 malaria-related deaths in 2000 (UNICEF/WHO, 2015; World Health Organization, 2019). These figures, however, have stalled over the last 3 years, indicating that the global response to malaria is not enough to achieve eradication. Numerous vaccines have been developed as additional tools to prevent malaria (Draper et al., 2018; Wilson et al., 2019; Yenkoidiok-Douti and Jewell, 2020). However, the lack of an effective vaccine, as well as the emergence of drug-resistant parasites and insecticide-resistant mosquitoes are important threats to recent gains and highlight the need for novel strategies to control malaria transmission and ultimately eliminate the disease.

*Plasmodium* fertilization takes place in the mosquito midgut and zygotes develop into ookinetes, which traverse the mosquito midgut epithelium and differentiate into oocysts (Phillips et al., 2017). The early stages of *Plasmodium* are mostly extracellular. Antiplasmodial effector molecules, such as host antibodies and complement present in the ingested blood, along with mosquito complement, come in direct contact with the parasite, resulting in dramatic parasite losses and a natural *Plasmodium* population bottleneck. As a result, mosquitoes naturally infected in endemic areas usually carry five or less oocysts (Smith et al., 2014). This makes mosquito stages attractive targets to disrupt malaria transmission.

Recently, several promising transmission-blocking vaccines (TBVs) to prevent transmission of malaria parasites from humans to mosquitoes have been reported. Most TBVs rely on host antibodies ingested during blood feeding, along with *Plasmodium* parasites, that bind to proteins on the surface of the parasite and block transmission by inhibiting parasite development (Sauerwein and Bousema, 2015; Schorderet-Weber et al., 2017). Over the last 20 years, a number of antigens, including Pfs230 (MacDonald et al., 2016; Marin-Mogollon et al., 2018; Scaria et al., 2019), Pfs48/45 (Theisen et al., 2014; Singh et al., 2017, 2019; Cao et al., 2018; Lennartz et al., 2018), and Pfs25 in *Plasmodium falciparum* as well as its ortholog Pvs25 in *Plasmodium vivax* (Miura et al., 2007; Lee et al., 2016; Blagborough et al., 2016; Brune et al., 2016; Leneghan et al., 2017; Parzych et al., 2018; Thompson et al., 2018; McLeod et al., 2019; Yusuf et al., 2019), have been identified as potential vaccine targets. Preclinical and clinical studies have shown that TBVs hold the promise to reduce malaria transmission and raise the prospect of providing an additional effective tool toward malaria eradication (Chichester et al., 2018; Sagara et al., 2018).

Most of the preclinical studies to test the efficacy of TBVs use a standard membrane feeding assay (SMFA) to determine the functionality of transmission-blocking antibodies (Sauerwein and Bousema, 2015). In this assay, *in vitro* cultured *P. falciparum* gametocytes are mixed with serum or purified antibodies and fed to laboratory-reared *Anopheles* mosquitoes through membrane feeders. The read-out of the SMFA is the proportion of infected mosquitoes (oocyst prevalence) and the percent reduction of oocyst density (transmission reducing activity, TRA) in experimental mosquitoes compared to controls (Sauerwein and Bousema, 2015; Draper et al., 2018). This assay is a useful tool to test vaccine efficacy, however, it relies on the availability and infectiousness of gametocytes produced in culture or obtained directly from infected hosts. Besides, it lacks the natural interaction of the mosquito with the host skin, immune cells, and coagulation factors that parasites would typically encounter in the host blood. As a result, it is hard to directly translate the efficacy of TBVs in pre-clinical studies to the outcomes of malaria transmission in the field. Thus, pre-clinical studies to test TBV candidates *in vitro* and *in vivo* are critical to assess their potential before proceeding to clinical trials.

We have recently shown that Pfs47, a paralog of Pfs48/45, is a promising TBV target (Alvaro et al., 2013; Canepa et al., 2018; Yenkoidiok-Douti et al., 2019), based on SMFA assays. *Plasmodium berghei*, a murine malaria model (Craig et al., 2012; Otto et al., 2014), also infects mosquitoes that transmit human malaria, including *A. gambiae*, a major vector in Africa (Cohuet et al., 2006; Dong et al., 2006). In this report, we describe the use of *P. berghei* to explore the *in vivo* potential of P47 as a malaria TBV target. We identified the region of the *P. berghei* P47 (Pbs47) that confers protection and conjugated the protective antigen to the bacteriophage AP205 virus-like particle (VLP) to enhance immunogenicity. AP205 is a bacteriophage coat protein that can be genetically fused to a protein adaptor “SpyCatcher” (Otto et al., 2014; Singh et al., 2017; Chichester et al., 2018). VLPs are non-infectious, self-assembling, multimeric proteins that resemble the structural organization and conformation of viruses (Brune et al., 2016; Frietze et al., 2016; Yenkoidiok-Douti et al., 2019). AP205-SpyCatcher is an engineered VLP that forms a covalent peptide bond *in vitro* when incubated with peptides tagged with a SpyTag (Zakeri et al., 2012; Li et al., 2014; Reddington and Howarth, 2015; Dovala et al., 2016; Govasli et al., 2019), making it possible to decorate the VLP with any antigen of interest. VLPs can traffic efficiently into draining lymph nodes and facilitate antigen uptake by antigen-presenting cells. Their repetitive structure induce efficient B-cells receptor clustering, thus improving the quantity and quality of the immune response generated (Hong et al., 2018). We found that VLP conjugation of the Pbs47 antigen enhanced immunogenicity and improved the TRA when IgG was passively transferred to infected mice. Our *in vivo* studies confirmed that a vaccine that targets a specific region of P47 is effective at disrupting malaria transmission.

## Results

### Antibodies to Full-Length Pbs47 Preferentially Target Domains 1 and 3, and Do Not Disrupt Malaria Transmission

**FIGURE 1:**
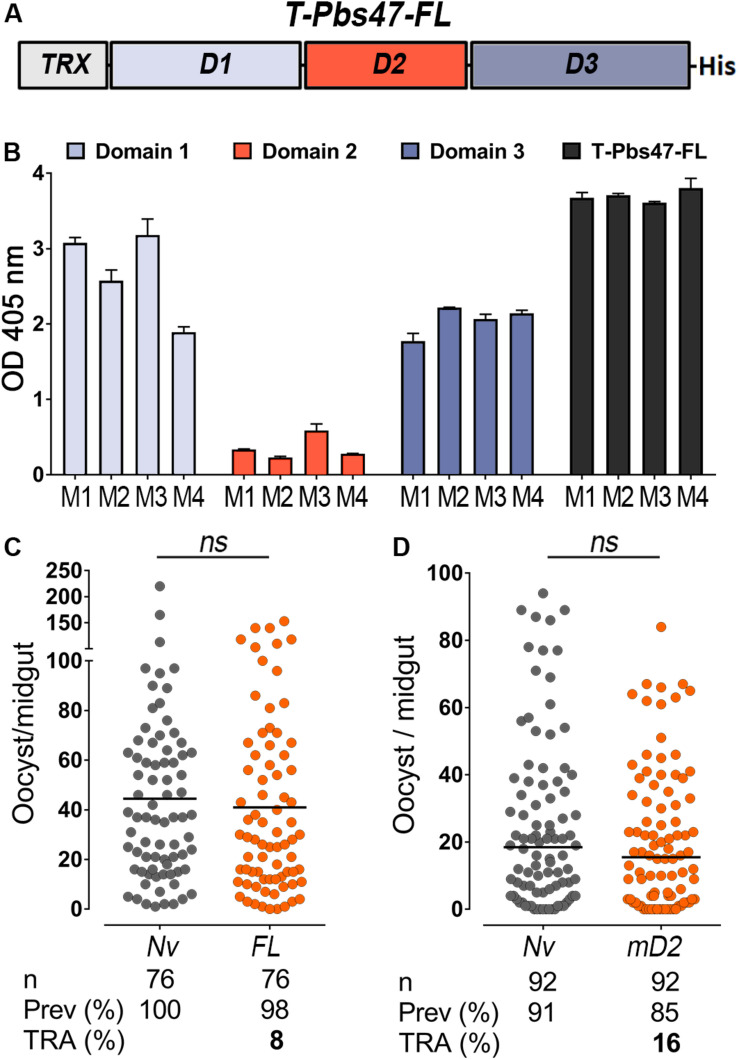
Immunoreactivity and transmission-reducing activity of total sera against modified T-Pbs47-FL and Pbs47-mD2. **(A)** Schematic representation of *Plasmodium berghei* T-Pbs47-FL fusion protein. Domains 1, 2, and 3 are represented to scale; His denotes a six-Histidine tag, “T” denotes Thioredoxin. **(B)** ELISA reactivity of sera from four mice immunized with T-Pbs47-FL against Pbs47 domains 1, 2, and 3; column and errors bars represent mean OD 405 ± standard deviation of two replicate assays. **(C)** TRA of T-Pbs47-FL and **(D)** Pbs47-mD2 following direct mosquito feeding on individual mouse. Each dot represents the number of oocysts in individual mosquito and the lines indicate the medians per group. Number of mosquitoes dissected (n); Infection prevalence (Prev); Transmission-reducing activity (TRA) as percent inhibition of infection intensity relative to naïve control mice. These data represent the overall result from *n* = 4 mice feeding. Medians were compared using the Mann–Whitney test: ns, no significant difference.

Pbs47 only shares 47% amino acid identity with its ortholog in *P. falciparum* (Pfs47) (Van Dijk et al., 2001; Molina-Cruz et al., 2017; Ukegbu et al., 2017; de Jong et al., 2020; Supplementary Figures 1A,B), but they have a similar structural organization with three 6-cysteine domains. Domains 1 and 3 have the canonical six-cysteine residues each, while domain 2 only has two cystines (Molina-Cruz et al., 2017). Recombinant full-length Pbs47 (without the predicted GPI anchor) was expressed as a fusion protein with thioredoxin at the N-terminus (T-Pbs47-FL) (Figure 1A and Supplementary Figures 2A,B) in *E. coli* and purified from the soluble fraction of the cell lysate using nickel affinity purification under native conditions (Supplementary Figure 2B). Individual domains 1 and 3 expressed without the thioredoxin fusion were insoluble and were purified from inclusion bodies using nickel affinity purification with a multistep refolding and buffer exchange protocol. Pbs47 domain 2 alone was toxic to *E. coli* and could only be expressed after the protein was modified by replacing the two cysteine residues in Pbs47 domain 2 by alanine (C202A and C232A). We will refer to this modified domain as Pbs47-mD2 (Supplementary Figures 3A–D). Similar toxicity was previously observed in *E. coli* when recombinant domain 2 of Pfs47 protein with the two cysteine residues was expressed (Canepa et al., 2018).

BALB/c mice were immunized intradermally with purified T-Pbs47-FL protein mixed with Magic Mouse^®^, a CpG-based adjuvant, to determine the immunogenicity of the antigen. Serum immunoreactivity to the full-length protein and individual domains was monitored by enzyme-linked immunosorbent assay (ELISA), at regular intervals throughout the immunization protocol for immunoreactivity. We found that antibodies from mice immunized with T-Pbs47-FL preferentially target domains 1 and 3, while immunoreactivity to domain 2 was very low (Figure 1B). This was also previously observed in mice immunized with full length Pfs47 (Canepa et al., 2018). The functional efficacy of the vaccine was evaluated by directly feeding *A. gambiae* mosquitoes on naïve or immunized mice infected with *P. berghei* parasites. The number of oocysts when mosquitoes were fed on the T-Pbs47-FL immunized group was not significantly different from those fed on naïve mice (Mann–Whitney, *p* = 0.3193) (Figure 1C), indicating that antibodies against domain 1 and 3 do not affect malaria transmission. Immunization with Pbs47-mD2 elicited a strong antibody response but also did not have a significant effect on the number of *Plasmodium* oocysts (Mann–Whitney, *p* = 0.3473 TRA, 16%) (Figure 1D).

### Antibodies Targeting a Specific Region of Pbs47 Domain 2 Reduce Malaria Transmission

**FIGURE 2:**
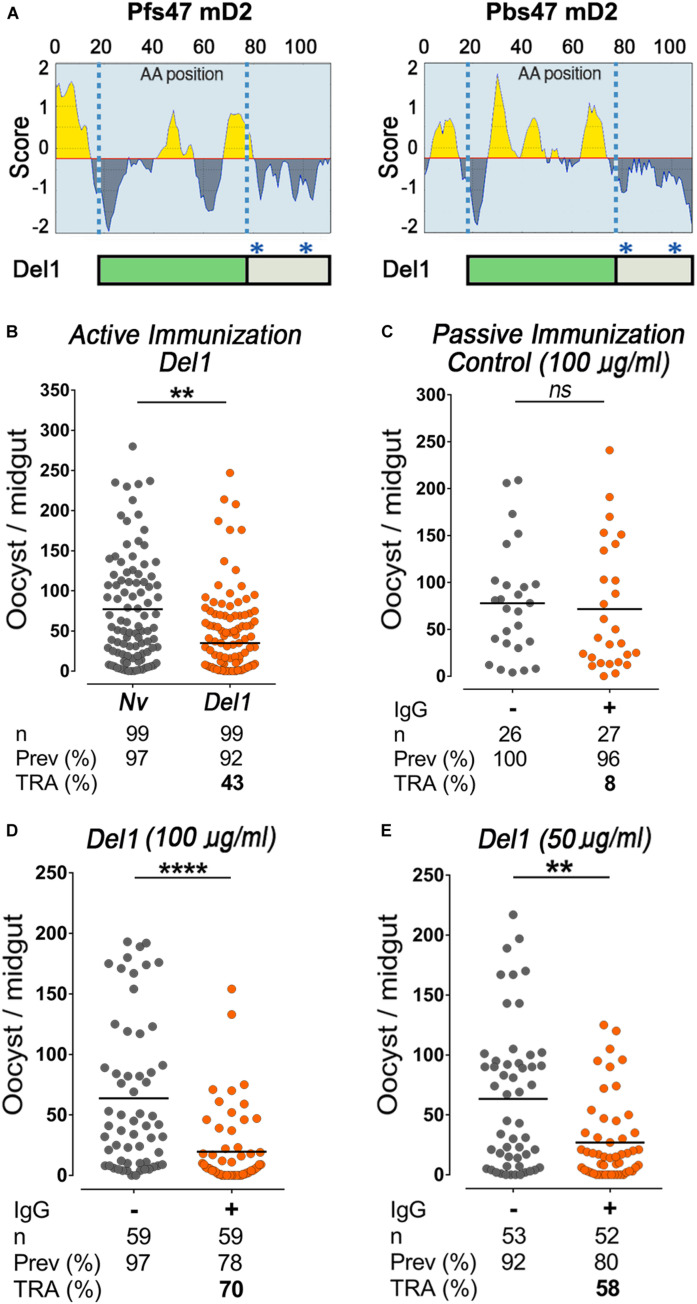
Epitope analysis of Pbs47-mD2, immunogenicity, and transmission-reducing activity of Del1. **(A)** Top, Linear B-cell epitopes prediction of Pfs47-mD2 (left) and Pbs47-mD2 (right) based on the Bepipred Program; Bottom, schematic representation of Pbs47-mD2 deletion, black asterisks indicate positions where cysteine residues were substituted with alanine. **(B)** Mean TRA of Del1 following direct mosquito feeding on mice, *n* = 4. **(C)** TRA following direct mosquito feeding on mice passively immunized with 100 μg/mL of naïve IgG. **(D)** TRA of Del1 following direct mosquito feeding on individual mice passively immunized with 100 μg/mL and **(E)** 50 μg/mL of Del1 purified IgG, respectively. Each dot represents the number of oocysts in individual mosquito and the lines indicate the medians per group. Number of mosquitoes dissected (n); Infection prevalence (Prev); Transmission-reducing activity (TRA) as percent inhibition of infection intensity relative to naïve control mice. This data represents the overall result from *n* = 2 replicates. Medians were compared using the Mann–Whitney test: ns, no significant difference; ns non-significant; **P < 0.01; ****P < 0.0001.

Previous studies with *P. falciparum* revealed that antibodies to the N-terminal region of Pfs47-mD2, where a linear epitope is predicted, are deleterious and enhanced infection in mosquitoes (Canepa et al., 2018). We also identified an N-terminus epitope in Pbs47-mD2 using a linear B-cell epitopes prediction software (Larsen et al., 2006; Figure 2A and Supplementary Figure 4A) and explored whether removing this epitope would enhance protection. A recombinant protein in which the N-terminal region of Pbs47-mD2 was deleted (Del1) was expressed, purified and used to immunize mice (Supplementary Figures 4B,C). The Del1 construct was immunogenic and the antibodies recognized T-Pbs47-FL, Pbs47-mD2, and Del1 (Supplementary Figures 4D,E). The level of infection in mosquitoes fed on mice immunized with Del1 was significantly lower than the naïve group (Mann–Whitney, *p* = 0.0014) with a 43% reduction in parasite infection intensity from a mean oocyts number of 77.1 to 47.0 (Figure 2B). In the *P. berghei* system, it is not possible to precisely match the parasitemia and gametocytemia of mice from the same experimental group, resulting in significant variation in the level of mosquito infection within the experimental groups (Naïve or Del1-immunize) (Supplementary Figure 5A). To overcome this problem, we established a passive immunization protocol in which we compared the TRA of mosquitoes fed on the same mouse, before and after intravenous injection of a known concentration of purified IgG from naïve or immunized mice (Supplementary Figure 5B). This method has the unique advantage that the same mouse serves as its own infection control, eliminating the variability in parasitemia or gametocytemia between mice.

To determine the concentration of antibody circulating in mice following passive immunization, we injected a set of naïve mice with anti-Del1 total IgG and collected their sera 30 min post-infusion. Antibody concentration in the sera after infusion was determined by ELISA, using the immunoreactivity of serial dilutions of purified IgG before intravenous (I.V.) infusion as reference. We established that I.V. injection of 5 μg of purified IgG per gram of mouse weight (5 μg/g) resulted in a final serum concentration of purified IgG of 100 μg/mL IgG (Supplementary Figure 5C).

To establish whether feeding a second mosquito cup on the same mouse could affect infectivity, *P. berghei*-infected mice were injected intravenously with 5 μg/g of weight of IgG purified from naïve mice (non-immunized). Infusion of IgG from naïve mice had no effect on oocyst density between mosquitoes fed before or after the IgG infusion (Figure 2C). In contrast, injection of purified IgG from mice immunized with Del1 significantly reduced the number of oocysts by 70% (Mann–Whitney, *p* < 0.0007) from a mean oocyts number of 63.7 to 19.6 (Figure 2D). Meanwhile at 50 μg/mL, anti-Del1 IgG inhibited parasite intensity by 58% (Mann–Whitney, *p* = 0.0014) from a mean oocyts number of 63.3 to 26.9 (Figure 2E). This dose-dependent response indicates that IgG directed against the Del1 region can reduce *Plasmodium* transmission to mosquitoes.

### Conjugation of Del1 to a VLP Enhances Immunogenicity, but Does Not Enhance Avidity and Results in a Modest Increase in Malaria Reducing Activity

**FIGURE 3:**
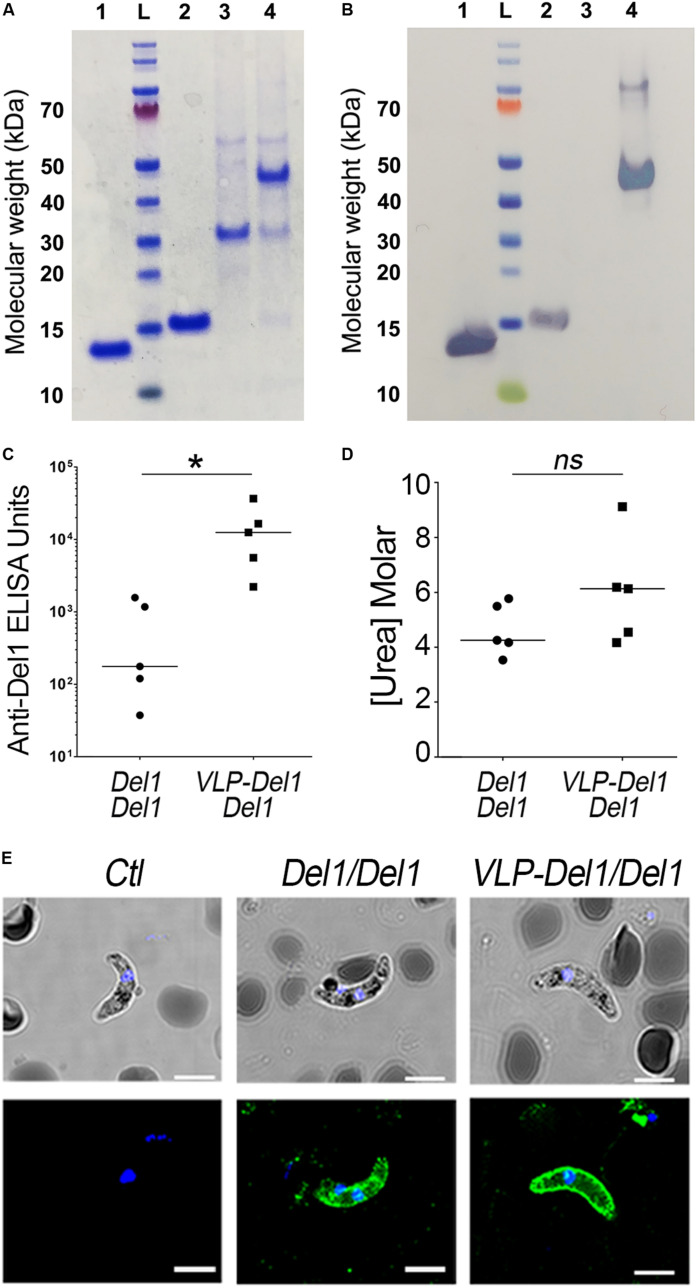
Expression, immunogenicity, and TRA of VLP-Del1. **(A)** Coomassie blue stained SDS SDS-PAGE and **(B)** Western Blot showing expression and conjugation of VLP particles. 1. Del1, L. ladder, 2. SpyTag-Del1, 3. SpyCatcher-AP205, 4. VLP-Del1 **(C)** ELISA showing the reactivity of serum collected from mice immunized intramuscularly with Del1 or VLP-Del1. **(D)** Antibody avidity was assessed by urea-displacement ELISA. The reduction in OD after incubating each antibody with increasing concentrations of urea (1–8 M) was compared to that after incubation without urea. The binding avidity index is shown, defined as the concentration of urea required to reduce the OD405 to 50% of that without urea **(E)** Immunofluorescence assay to detect native Pbs47 on Ookinetes. As a negative control, parasites were stained with antibodies from naïve mice (left). Parasites were stained with antibodies derived from mice immunization with Del1 and VPL-Del1 in green. DNA was stained with DAPI (blue); Antibody binding was detected by Alexa Fluor 488-conjugated goat anti-mouse or anti-rabbit IgG. DIC view (top) and a merge of DAPI and antibody staining (bottom) are shown. The staining was imaged using a confocal microscope; Scale bar: 2 μm. Medians were compared using the *t*-test: ns, non-significant; *P < 0.05.

Because the Del1 peptide is small (13 kDa), we explored whether we could enhance its immunogenicity by conjugating it to a VLP carrier (AP205). A recombinant Del1 protein with a “SpyTag” was expressed, purified and covalently linked to the surface of a AP205-VLP, using the AP205-SpyCatcher:SpyTag platform (Figures 3A,B). We have previously shown that, for *P. falciparum* Pfs47, a 95% coupling efficiency was achieved when SpyTag-Pfs47 was mixed with AP205-SpyCatcher at a 1:1 molar ratio (Yenkoidiok-Douti et al., 2019). Thus, AP205-SpyCatcher with Pbs47 SpyTag-Del1 were mixed at 1:1 molar ratio and this resulted in a covalently bound protein of the expected size (48 kDa) under denaturing and reducing conditions (Figure 3A). Western blot analysis showed that the conjugated VLP reacted strongly with anti-Del1 total antibodies, while, as expected, no signal was observed for the unconjugated SpyCatcher-AP205 VLP (Figure 3B). Furthermore, no free uncoupled SpyTag-Del1 could be detected after conjugation, confirming the high efficiency of this reaction (Figure 3B). Negative-staining transmission electron microscopy (TEM) confirmed that the AP205 particles conjugated with Del1 antigen are homogenous and have the expected size (∼20–30 nm) (Supplementary Figure 6A).

We have previously shown that prime-boost immunization with *P. falciparum* VLP-Pfs47 elicits a very strong immune response to the VLP carrier, and a better TRA was obtained when prime immunization with VLP-Pfs47 was followed by a boost with Pfs47 monomer (Yenkoidiok-Douti et al., 2019). Thus, the immunogenicity of antibodies generated with two different immunization strategies were evaluated: (1) Prime-boost with *P. berghei*, Del1 monomer antigen (Del1/Del1) and (2) VLP-Del1 priming followed by a boost with Del1 monomer (VLP-Del1/Del1). For both groups, mice received 1 μg Pb-Del1 antigen equivalents either as monomer or as a VLP. Antibody response of the VLP-Del1/Del1 group was significantly higher than the Del1/Del1 (Mann–Whitney, *p* = 0.0482), with a 72-fold increase in the median anti-Del1 ELISA Units (Figure 3C). However, the median avidity index of serum antibodies, defined as the concentration of urea required to reduce the OD405 to 50% of that without urea, was not significantly different between the two immunization schemes (Mann–Whitney) (Figure 3D). It is noteworthy, that there are important differences in the avidity index between individual mice. For example, anti-Del1 antibodies from a mouse of the VLP-Del1/Del1 immunization group had very high avidity, as the binding was more than 50% of the control at the highest concentration used to dissociate antibody binding (8M Urea), and the regression model estimated an avidity index of 9M Urea (Figure 3D). We confirmed that total IgG against Del1 from mice immunized with either Del1/Del1 or VLP-Del1/Del1 both recognize native Pbs47 on the parasite’s surface (Figure 3E).

**FIGURE 4:**
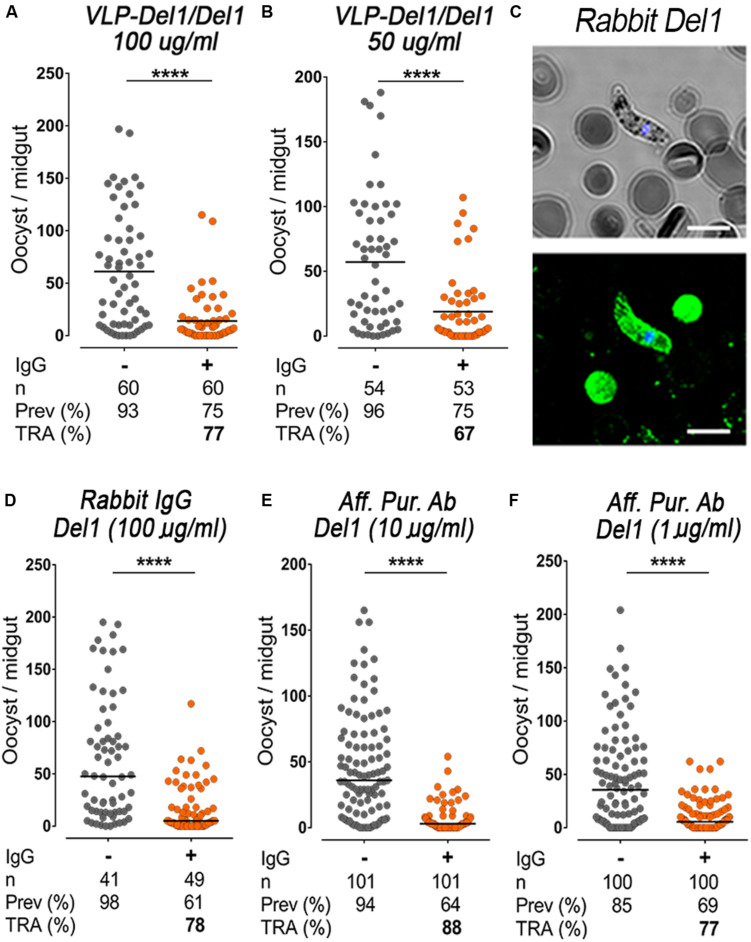
Functional characterization of total sera against Del1 recombinant protein. **(A)** TRA of VLP-Del1 following direct mosquito feeding on mice passively immunized with 100 μg/mL and **(B)** 50 μg/mL of purified IgG, respectively. **(C)** Immunofluorescence assay to detect native Pbs47 on Ookinetes. As a negative control, parasites were stained with antibodies from naïve mice (left). Parasites were stained with antibodies derived from rabbit immunization with Del1 in green. DNA was stained with DAPI (blue); Antibody binding was detected by Alexa Fluor 488-conjugated goat anti-mouse or anti-rabbit IgG. DIC view (top) and a merge of DAPI and antibody staining (bottom) are shown. The staining was imaged using a confocal microscope; Scale bar: 2 μm. **(D)** TRA of rabbit Del1 IgG following direct mosquito feeding on mice passively immunized with 100 μg/mL. **(E)** TRA of rabbit Del1 affinity purified IgG following direct mosquito feeding on mice passively immunized with 10 μg/mL and **(F)** 1 μg/mL of purified IgG, respectively. Each dot represents the number of oocysts in individual mosquito and the lines indicate the medians per group. Number of mosquitoes dissected (n); Infection prevalence (Prev); Transmission-reducing activity (TRA) as percent inhibition of infection intensity relative to naïve control mice. This data represents the overall result from *n* = 2 replicates. Medians were compared using the Mann–Whitney test: ****P < 0.0001.

Total IgG purified from pooled sera of VLP-Del1/Del1 immunized mice was infused intravenously to *P. berghei*-infected recipient mice at concentrations of 100 and 50 μg/mL. A significant reduction in the number of oocysts was observed with both doses (Mann–Whitney, *p* < 0.0001) with TRA of 77% at 100 μg/mL (from a mean oocyts number of 61.1 to 13.9) and of 67% at 50 μg/mL (from a mean oocyts number of 57.1 to 18.8) (Figures 4A,B). At a concentration of 50 μg/mL, the TRA after VLP-Del1/Del1 immunization was modestly stronger (67%) (Figure 4B) when compared with the Del1/Del1 monomer (58%, from a mean oocyts number of 63.3 to 26.9) (Figure 2E).

### Affinity Purified Del1-Specific Antibodies Strongly Reduce Parasite Loads in Mosquitoes

It is difficult to compare the efficiency of different antigens based on IgG purified from immunized mice, as one does not know the proportion of IgG antibodies specifically directed to the antigen of interest (Supplementary Figure 6B). A rabbit was immunized with Del1 to obtain enough serum to purify Del1-specific antibodies. These generated antibodies can detect native Pbs47 protein on the surface of ookinetes when tested by immunofluorescence assay (Figure 4C). Furthermore, infusion of rabbit IgG to *P. berghei*-infected mice induced 78% inhibition of oocyst intensity (from a mean oocyts number of 63.2 to 14.5) at 100 μg/mL (Figure 4D). Del1-specific antibodies were purified using affinity columns with immobilized Del1 antigen, as previously described (Menon et al., 2018). Affinity-purified antibodies were functional, as purification increased the ELISA immunoreactivity to Del1 by 10-fold (Supplementary Figure 7). Intravenous infusion of affinity-purified antibodies significantly reduced oocyst density by 88% (from a mean oocyts number of 46.0 to 5.6) and 77% (from a mean oocyts number of 44.6 to 10.4) respectively at doses as low as 10 and 1 μg/mL (Figures 4E,F). This demonstrates that antibodies against Del1 are functional and potent *in vivo.*

## Discussion

It is clear that reducing the rate of re-infection in humans will be critical to eradicate malaria (Blagborough et al., 2013), and that the natural population bottlenecks in mosquito stages of *Plasmodium* are vulnerable points to disrupt the parasite’s life cycle and prevent disease transmission (Smith et al., 2014; Sauerwein and Bousema, 2015). Recent studies demonstrated the potential of *P. falciparum* P47 (Pfs47) as a TBV candidate (van Dijk et al., 2010; Arredondo and Kappe, 2017) based on SMFAs, a robust *in vitro* assay that tests the efficiency of antibodies to different vaccine targets. In this study, we evaluated the transmission-reducing activity of a P47-based vaccine *in vivo* using the *P. berghei* murine model. We found that although Pbs47 and Pfs47 have limited sequence conservation, they share remarkable similarities in their immunogenicity and in the regions where antibody binding affects transmission (Canepa et al., 2018). For example, in both cases immunization with full-length P47 proteins induced antibodies primarily against domain 1 and domain 3 (Figure 1B), but not domain 2, and antibodies that target an immunodominant epitope in the N-terminus of domain 2 were deleterious. Furthermore, for both antigens, only antibodies targeting the same specific region of domain 2 confer transmission-reducing activity (Figure 2A).

A limitation of the *P. berghei* model is the inherent variability on how well a particular mouse infects mosquitoes. We found that passive infusion of purified IgG from an immunized mouse made it possible to directly compare the infectivity of the same mouse before and after the antibodies were infused. The dose-dependent efficiency of the infused IgG after immunization indicates that inducing a sustained high level of circulating antibodies is critical to elicit a strong TRA. Immunization with the Pbs47-Del1 antigen conjugated to AP205-VLP (VLP-Del1/Del1) enhanced immunogenicity, but the avidity of the antibodies was not significantly different from mice that received a prime-boost with the Del1 monomer (Del1/Del1). Although we were able to enhance the immune response, when the level of purified IgG was adjusted, the TRA after VLP-Del1/Del1 immunization only increased modestly compared to Del1/Del1 mice. However, the 72-fold increase in ELISA immunoreactivity in the VLP-Del1/Del1 relative to Del1/Del1 is expected to prolong the time that immunized mice maintain antibody levels above the threshold to prevent mosquitoes from becoming infected with *Plasmodium*. The efficiency of antigen-specific IgG was tested with affinity-purified anti-Del1 antibodies and confirmed that Pbs47-specific antibodies induce a strong TRA (77%) at doses as low as 1 μg/mL (Figure 4F).

This study provides clear *in vivo* evidence that, as with other targets (Cockburn and Seder, 2018), the ability of a P47-based TBV to disrupt malaria transmission depends on the potency and duration of the antibody response elicited by the vaccine. More specifically, TBVs must induce sustained high antibody titers of high affinity binding antibodies that target key epitopes of the parasite. This is the first report demonstrating striking functional similarities between two P47 orthologs of evolutionarily distant parasites as transmission-blocking vaccine antigens.

## Materials and Methods

### Recombinant Protein Expression and Purification

Coding DNA for Pbs47 (PBANKA_1359700) full-length Ile22 to Gly380 (T-Pbs47-FL) was inserted into a modified pET32 plasmid such that Pbs47 protein contains an N-terminal Thioredoxin and a C-terminal hexa-histidine tag. Cloning was performed with In-Fusion HD kits (Clontech) and inserts were confirmed by Sanger sequencing. The Thioredoxin-Pbs47 (T-Pbs47-FL) construct (Ile22 to Gly380) was codon optimized for *Escherichia coli* expression. pET32-T-Pbs47-FL constructs were transformed into *E. coli* BL21 (DE3) pLysS RosettaTM. For protein expression, the transformed *E. coli* cells were expanded at 37°C, shaking at 220 rpm and grown to an OD600 of 0.6–0.8. The protein expression was induced at lower temperature (30°C) for 4 h with 1 mM isopropyl β-D-thiogalactopyranoside (Sigma). The rT-Pbs47-FL was present in the soluble fraction of the cells lysate; therefore, we performed purification by nickel affinity purification chromatography (Thermo Fisher Scientific) under native conditions. The purified protein was dialyzed in a buffer containing 20 mM HEPES pH 7.5, 250 mM NaCl, 0.1 mM EDTA, 5 mM MgCl2.

The construct for the modified Pbs47 domain 2 protein (mD2) (K129-I237) was synthetized replacing the two cysteine residues for alanines (C202A and C232A). A hexahistidine tag was added at the C-terminal and the construct was subcloned into the pET17b plasmid. Pbs47 Domain 1 and 3 were designed using the same His-tag and were cloned using the same pET17b expression vector. Pbs47 domain 2 was used as template to generate the N-terminal deletion construct (Del1) (Ile152 to Ile237) using PCR and was subcloned into pET17b. Cultures of Pbs47-D1, D2, D3, and Del1 were induced for expression for 3 h at 37°C with 1 mM isopropyl β-D-thiogalactopyranoside (Sigma). The expressed proteins localized to inclusion body pellets and were resuspended in 8M urea solution to be purified by in-column HPLC nickel affinity purification/refolding chromatography (Thermo Fisher Scientific). Proteins were then dialyzed against HEPES-buffered saline (20 mM HEPES pH 7.5, 150 mM NaCl) containing 2% glycerol. All gel images derive from the same experiment and were processed in parallel.

### Cloning of AP205-SpyCatcher and SpyTag-Del1

SpyCatcher-AP205 was synthesized by BioBasic and was cloned into pET17b vectors with the following order: ΔN1SpyCatcher (Tissot et al., 2010; Brune et al., 2016; Yenkoidiok-Douti et al., 2019), (GSG)_3_ linker, codon-optimized AP205 coat protein 3 (Yenkoidiok-Douti et al., 2019), and C-terminal His_6_ (Supplementary Figure 1A). SpyTag-Del1 was cloned into pET17b with the following order: SpyTag (Tissot et al., 2010), (GSG)_3_ linker, Del1, and C-terminal His_6_ (Supplemenary Figure 2A). Cloning was performed with In-Fusion HD kits (Clontech) and inserts were confirmed by Sanger sequencing.

### Expression of SpyCatcher-AP205

SpyCatcher-AP205 was expressed by adapting a previously described protocol (Brune et al., 2016; Yenkoidiok-Douti et al., 2019). Briefly, BL21(DE3) pLysS chemically competent *E. coli* cells (Thermo Fisher Scientific) were transformed with pET17b-SpyCatcher-AP205 and inoculated onto LB agar plates with 100 μg/mL ampicillin and 34 μg/mL chloramphenicol. The plates were incubated O/N at 37°C. Ten transformed BL21 colonies were picked and cultured in 10 separate tubes containing 4 mL LB supplemented with 100 μg/mL ampicillin and 34 μg/mL chloramphenicol O/N at 37°C. These cultures were added to 1L of LB broth supplemented with 100 μg/mL ampicillin and 34 μg/mL chloramphenicol and shaken at 160 rpm at 37°C until OD600 was 0.6–0.9. Expression was induced with 1 mM IPTG for 6 h at 30°C. The culture was then centrifuged at 4000 × g for 20 min at 4°C. The resulting pellet was resuspended with 50 mL cold PBS, spun again for 10 min, and left frozen at −20°C O/N.

### Purification of SpyCatcher-AP205

Soluble AP205-SpyCatcher was purified following a previously described protocol (Yenkoidiok-Douti et al., 2019). Pellets from 1 L of culture were resuspended at RT in 30 mL HEPES-buffered saline (HBS) lysis buffer (20 mM HEPES, 150 mM NaCl, 0.1% triton X-100, 0.1% Tween 20) and incubated on ice for 10–15 min. The pellets were sonicated 4× for 30 s–2 min and the lysates were then spun twice at 15,000 × g for 20 min at 4°C. The supernatants were filtered through 0.45 μm SFCA filters (Thermo Fisher Scientific) and dialyzed O/N in the same solution using a 300 kDa cut-off membrane. To bring lysates to 50 mM imidazole, appropriate volumes of Ni-NTA elution buffer (20 mM HEPES, 150 mM NaCl, and 1M imidazole) were added to the filtered lysates, along with 500U benzonase (Sigma Aldrich) and 3 mL HisPur Ni-NTA resin (Life Technologies). The lysates were left slowly rocking at 4°C for 20–25 min and were then added to two 12 mL chromatography columns (BioRad) for gravity filtration. The resin was washed with 40–50 mL of 100 mM imidazole buffer (20 mM Hepes, 150 mM NaCl, 100 mM imidazole) followed by an additional 50 mL wash in the same washing buffer containing 0.1% triton X-114 (Sigma) to remove endotoxin. The proteins were eluted into 500 μL fractions with 2M imidazole, 20 mM Hepes, 150 mM NaCl, and 0.1% Tween 20. Elutes were dialyzed with a 300 kDa MWCO Spectrum^TM^ Spectra/Por^TM^ Float-A-Lyzer^TM^ G2 Dialysis Device (Thermo Fisher Scientific) in 20 mM HEPES, 150 mM NaCl, and 0.1% Tween 20. SpyCatcher-AP205 protein underwent a final dialysis with the same 300 kDa MWCO dialysis device (Thermo Fisher Scientific) into PBS pH 4.5 to equilibrate the buffer with that of the SpyTag-Del1 protein.

### Expression of SpyTag-Del1

BL21(DE3) pLysS chemically competent *E. coli* cells were transformed with pet17b-SpyTag-Del1. One colony was cultured in 4 mL of LB media with ampicillin and chloramphenicol O/N at 37°C and this culture was added to 100 mL of fresh LB media with antibiotics, as described above. Expression was induced with 1 mM IPTG for 4 h at 37°C at OD600 ∼0.4–0.6. The culture was centrifuged at 4000 × g for 20 min at 4°C and the pellet frozen at −20°C O/N.

### Purification of SpyTag-Del1

The SpyTag-Del1 pellet was resuspended in 20 mL PBS and sonicated 4 times for 30 s to 2 min. Lysates were then centrifuged at 35000 × g for 15 min at 4°C. Resulting pellets were resuspended in PBS + 1% triton X-100 and sonicated 4 times for 30 s to 2 min. The suspension was again centrifuged at 35,000 × g for 15 m at 4°C. The resulting inclusion body pellet was resuspended in PBS 6M Urea, 1M NaCl, and 20 mM imidazole and left on ice for 30 min to 2 h. The suspension was then pelleted at 35,000 × g for 15 min at 4°C.

The supernatant was added to 1 mL HisPur Ni-NTA resin (Life Technologies) that had been equilibrated in a 12 mL chromatography column (BioRad). The resin was washed with 25 mL equilibration buffer (PBS 6M Urea, 1M NaCl, 20 mM imidazole) and subsequently washed with 10 mL washing buffer (PBS 2M Urea, 1M NaCl, 20 mM imidazole). The protein was eluted into 1 mL fractions with PBS 2M Urea, 1M NaCl, 300 mM imidazole. Elutes were dialyzed in PBS pH 4.5.

### SpyCatcher-AP205:SpyTag-Del1 Conjugation

Reactions occurred at room temperature for 3 h in a total volume of 25 μL. SpyTag-Del1 and SpyCatcher-AP205 were mixed at a 1:1 molar ratio and PBS pH 4.5 was used to bring the reaction volume to 25 μL. The reaction was stopped by adding 1× LDS sample buffer and NuPAGE Sample Reducing Agent (Thermo Fisher Scientific) and was analyzed by SDS-PAGE.

### SDS-PAGE

All proteins were boiled under reducing conditions in 1× LDS sample buffer (Thermo Fisher Scientific) and were separated using 4–12% NuPAGE Bis-Tris protein gels (Thermo Fisher Scientific). Gels were stained with Coomassie Blue. Protein concentrations were determined using Bradford protein assay (Thermo Fisher Scientific).

### Western Blot

Protein gels were transferred onto nitrocellulose membranes, which were placed in blocking solution containing 5% powdered milk (Sigma-Aldrich), 50 mM Tris-Cl, 150 mM NaCl, and 1% Tween 20 (TBST) at room temperature for 2 h. Recombinant proteins were detected using primary antibodies against the 6×-His epitope tag (Thermo Fisher Scientific cat # MA1-21315) or animal sera at 1:1000 dilution. The membranes were washed three times with TBST and incubated with goat anti-mouse or anti-rabbit secondary antibodies conjugated to alkaline phosphatase at 1:5000 dilution. Proteins were visualized using Western Blue Stabilized Substrate to detect alkaline phosphatase activity (Promega cat # S3841).

### IgG Purification

To perform passive immunization, mouse sera were pooled and the IgG purified, as previously described (Menon et al., 2018). Sera was collected from mice 2 weeks post-boost immunization and pooled within each group irrespective of individual antibody titer. Total IgG was purified using Protein G columns (Pierce, United States) and buffer exchanged to 1× PBS pH 7.4.

For affinity purification, Del1 antigens were immobilized in a column containing NHS-activated sepharose beads (GE Healthcare, #17-0906-01). A sample of total IgG antibody was diluted in a binding buffer (50 mM Na_2_ HPO_4_, pH 7.0) and passed through the column. Unspecific antibodies were washed using a washing buffer containing (0.1M acetic acid, 0.5M NaCl, pH 4). Del1-specific antibodies were eluted using an elution buffer (0.1M glycine, pH 2.7), dialyzed in PBS, and stored at −80°C until needed.

### Transmission Electron Microscopy (TEM)

SpyCatcher-AP205 (10 μL at a concentration of 0.2 mg/mL) were applied to 200 mesh copper grids with a thin carbon film (Electron Microscopy Sciences) and were glow discharged for 1 m before negative staining with 2% aqueous uranyl acetate followed by a brief water rinse. Grids were imaged at 80 kV with Hitachi HT-7800 transmission electron microscope equipped with an XR81-B CCD detector camera (AMT) set at a pixel size of 0.52 nm (nominal magnification of 30,000×). Particle size was modeled with IMOD (Kremer et al., 1996) and size distribution plot was made using GraphPad Prism software.

### Animals

All animal procedures were performed according to protocols approved by the NIAID and NIH Animal Care and Use Committee. Five to eight weeks old naïve, female Balb/c mice were purchased from Charles River (Germantown, MD) and maintained at a facility at the NIH.

Public Health Service Animal Welfare Assurance #A4149-01 guidelines were followed according to the National Institutes of Health Animal (NIH) Office of Animal Care and Use (OACU). These studies were done according to the NIH animal study protocol (ASP) approved by the NIH Animal Care and User Committee (ACUC), with approval ID ASP-LMVR5.

### Mice Immunization

Groups (*n* = 4 or 5) of female BALB/c mice (Charles River, Wilmington, MA, United States) were immunized intramuscularly in the rear thigh with a 50 μL vaccine containing a total of 1 μg of Pbs47 antigen or 20 μL vaccine containing 1 μg via intradermal injections in the dorsal ear skin, either as monomer or conjugated as a VLP-Pbs47 particle formulated with Magic Mouse^®^ adjuvant (Creative Diagnostics # CDN-A001). The immunized mice were boosted 4 weeks after the primary injection with the same dose of vaccine in alternative limbs or ears. Blood was collected on day 0 (Pre-immune sera) and 2 weeks after each subsequent immunization for *in vitro* analysis. The blood samples were allowed to clot at room temperature for 15 min before centrifugation at 13,000 × g in a benchtop centrifuge and serum was removed for testing. Endotoxin levels were quantified using Pierce LAL Chromogenic Endotoxin Quantitation Kit (Thermo Fisher Scientific) and were below 1 Unit/mL.

### Enzyme-Linked Immunosorbent Assay (ELISA)

Flat-bottom 96-well ELISA plates (Immunolon 4; VWR cat # 62402-959) were coated with 100 ng/well of recombinant protein diluted in coating buffer (15 mM Na_2_CO_3_, 35 mM NaHCO_3_, pH 9.6) overnight at 4°C. Plates were washed three times with TBST and blocked with general block ELISA blocking buffer (ImmunoChemistry cat # 640) for 2 h at 37°C. Animal sera were diluted in a 1:1 ratio buffer containing blocking buffer and TBST, added to the antigen-coated wells and incubated for 2 h at 37°C. The plates were then washed and incubated with goat anti-mouse or anti-rabbit immunoglobulin G conjugated to alkaline phosphatase (Seracare cat # 5220-0303) secondary antibodies in 1:2500 dilutions for 2 h at 37°C. The plates were washed again and detection was performed using 100 μL/well of p-nitrophenyl disodium phosphate solution (Sigma 104 phosphatase substrate; 1 tablet per 5 mL of coating buffer). After 20 min, absorbance was read at 405 nm with VersaMax ELISA plate reader. Titers were determined at OD reading of 0.5 by linear regression analysis with GraphPad prism software.

Antibody avidity was assessed by urea-displacement ELISA in which the reduction in OD after incubation of each antibody with increasing concentrations of urea (1–8 M) was compared to the OD after incubation without urea. A similar ELISA protocol was followed, as described above, except for a 15 min incubation step with 0, 1, 2, 4, 6, 8 M Urea that was introduced between the primary and secondary antibody incubations. The relative levels of bound antibody were determined using the standard ELISA procedure. The binding avidity index, defined as the concentration of urea required to reduce the OD405 to 50% of that without urea was calculated for each antibody using a sigmoidal dose response (non-linear) regression analysis.

### Immunofluorescence Assay (IFA)

Midguts from 25 mosquitoes were collected in PBS between 19 and 21 h after feeding on a *P. berghei* infected mouse. The midguts were grinded in an Eppendorf tube containing 1 mL PBS and washed five times in PBS. Between each wash, the tube was centrifuged at 2500 × g for 5 min. After the last wash, the pellet containing some red blood cells and ookinetes were resuspended in 1 mL PBS. Hundred microliter of the solution was allowed to settle on 10 wells glass slides for 30 min. The slides were fixed with 4% paraformaldehyde (PFA) plus 0.01% glutaraldehyde in PBS for 20–25 min, prior to blocking with 2% BSA and 0.1% gelatin in PBS. Mouse anti-sera or purified antibody were diluted to 1:100 and 1:500, respectively, and used to stain the ookinete for 2 h at room temperature. Secondary Alexa Fluor 488 donkey anti-rabbit or anti-mouse antibodies (Abcam cat # ab150105) were used at 1:2500 dilution for 2 h at room temperature and counter stained with DAPI. Slides were imaged using a Leica TCS SP8 DMI6000 inverted fluorescence confocal microscope (Leica Microsystems) at 60× magnification. The images were processed by deconvolution using the Huygens software and visualized using Imaris 8.3.1 (Bitplane AG).

### Passive Immunization

Six- to eight-week-old female BALB/c mice infected with GFP *P. berghei* parasites (ANKA GFPcon 259cl2) were randomized into two groups (*n* = 2 per group). The mice were anesthetized with a xylazine-ketamine cocktail injected intraperitoneally, fed to mosquitoes for 15 min, and allowed to regain consciousness over 2 h. Subsequently, experimental group mice were injected intravenously in the tail vein with purified total IgG (dialyzed in PBS) against *Plasmodium berghei* surface protein (Pbs47). The amount of antibody injected was based on mouse weight, assuming the average total blood volume of a mouse is about 80 mL/kg (The Jackson Laboratory, 2020). Control mice only received PBS or naïve mouse antibody via the same injection route. Thirty minutes after injection of the antibodies, the mice were anesthetized, and mosquitoes were allowed to feed on them for 15 min. Unfed mosquitoes were separated after 1 h of feeding and blood fed mosquitoes (typically 25–30 per cup) were maintained in the insectary at 19°C, 80% relative humidity. Blood samples were collected from the mice after the second feeding to confirm the presence of injected antibodies via ELISA.

### Immunized Mice Challenge

Two weeks after the final immunization, each mouse was infected intraperitoneally (I.P.) with 10^7^ *P. berghei*-infected erythrocytes taken from a third-passage blood-stage infection. During the infection, the parasitemia (% infection) of each mouse was examined daily in Giemsa-stained thin-film blood smears. The parasites were counted from at least 10 fields at the regions of the slides where the cells were equally distributed.

### Mosquito Feeding

Female *Anopheles gambiae* mosquitoes (G3) collected from a single emergent batch of a highly inbred line showing consistently high susceptibility to *P. berghei* infection were caged (30–50 per cage) and kept on water diet 12 h before blood feeding. Two days after mice parasite inoculation, parasitemia was counted on a Giemsa stained blood smear and exflagellation of male gametocytes was checked by addition of a drop of exflagellation media (RPMI 1640 medium containing 50 mg/L hypoxanthine, 2 g/L sodium bicarbonate, 50 U/mL penicillin, 50 mg/L streptomycin, Xanthurenic acid to a final concentration of 100 μM, supplemented with 20% heat inactivated fetal bovine serum (v/v), and 30 U/mL heparin, pH 8.3) to a drop of tail blood. Once mice reached 3% parasitemia, they were anesthetized and offered to a separate cage of mosquitoes for 15 min. Unfed mosquitoes were removed, and the remainder (usually 90–95% of the initial amount) were kept at 19°C and 80% relative humidity for 7 days to allow oocyst development.

### Determination of Percent Transmission (%Tm) and Transmission Reducing Activity (TRA)

Surviving mosquitoes (typically 85–90% of those fed) were dissected after 7 days. Mosquito midguts were dissected and fixed in 4% (v/v) PFA in PBS for 20 min at room temperature, washed twice for 5 min in PBS and mounted on glass slides in Vectashield^®^ (VectorLabs). The guts were observed under a Leica DM6000 epifluorescence microscope (Leica microsystem) using 10× magnification. The microscope is equipped with a Leica DFC 450C color camera, and a Leica DFC 345 cooled monochrome camera. The number of oocysts and the number of infected mosquitoes (prevalence) was scored and reported as the arithmetic mean of the three subgroups + the standard error of the mean. Percent transmission (%Tm) and percent blockade (TRA) were calculated from the following equations:

$$\begin{array}{l} \%Tm=\frac{\begin{array}{c} Arithmetic\;mean\;number\;of\;oocysts\;per\\ midgut\;in\;experimental\;group \end{array}}{\begin{array}{c} Arithmetic\;mean\;number\;of\;oocysts\;per\\ midgut\;in\;conrol\;group \end{array}}\times 100\\ TRA(\%)=100-Tm \end{array}$$

### Statistical Analysis

Statistical analyses were performed using Prism 8.3.0 (GraphPad). The Mann–Whitney U-test for non-normal distributions was used to determine statistical significance in parasite loads between two groups. To determine whether there was a difference in the quality of induced antibodies among two groups, an unpaired Student’s *t*-test (two-tailed) was used. One-way analysis of variance with Tukey post-test was used to compare differences between more than two groups. *P* < 0.05 was considered significant.

## Data Availability Statement

All datasets presented in this study are included in the article/Supplementary Material.

## Ethics Statement

The animal study was reviewed and approved by the Public Health Service Animal Welfare Assurance #A4149-01 guidelines were followed according to the National Institutes of Health Animal (NIH) Office of Animal Care and Use (OACU). These studies were done according to the NIH animal study protocol (ASP) approved by the NIH Animal Care and User Committee (ACUC), with approval ID ASP-LMVR5.

## Author Contributions

LY-D, GC, and AB performed the research. LY-D and CB-M wrote the manuscript. All authors reviewed the final manuscript, designed the research, and analyzed the data.

## Conflict of Interest

The authors declare that the research was conducted in the absence of any commercial or financial relationships that could be construed as a potential conflict of interest.
