# Supplementary material for: In vivo Characterization of Plasmodium berghei P47 (Pbs47) as a Malaria Transmission-Blocking Vaccine Target
Source: Front Microbiol. 2020 Jul 3;11:1496. doi: 10.3389/fmicb.2020.01496 (PMC7348136; doi:10.3389/fmicb.2020.01496)
Supplement: Supplementary file 1 [file Data_Sheet_1.PDF]

Supplementary Material

Characterization of *Plasmodium berghei* P47 as a Malaria Transmission-Blocking Vaccine Target

Lampouguin Yenkoidiok-Douti[1,2], Gaspar E. Canepa[1], Ana-Beatriz F. Barletta[1], Carolina Barillas-Mury[1]

***Correspondance**

Dr. Carolina Barillas-Mury

cbarillas@niaid.nih.gov

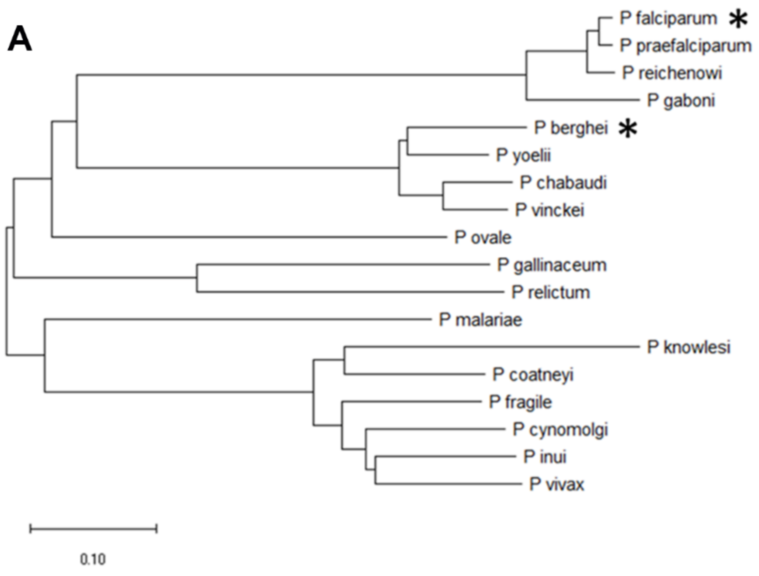

B

Score	Expect	Method	Identities	Positives	Gaps
371 bits(952)	5e-129	Compositional matrix adjust.	206/436(47%)	294/436(67%)	22/436(5%)

```
P. berghei     8    SIIVFYL---IKGYLSYIIFPNGYVCDFKFNPLVNVLPSINTTGDI-EEVGCTINNPSLS  63
                    +II+FY    +K  +S ++    YVCDF FNPL NV P++  + +I EEVGCTINNP+L
P. falciparum  11   NIILFYFFLWVKKSISELLSSTQYVCDFYFNPLTNVKPTVVGSSEIYEEVGCTINNPTLG  70

P. berghei     64   DYIALVCPKKNYNDYEHMEKVPSKCFSSNLYSPYKSEDSAHKLEELKIPEKYSISKDFSD  123
                    D+I L+CPKKN  D+ ++E VP+ CF S+LYS YK++ SA+ LE+L I +KY+I+  FSD
P. falciparum  71   DHIVLICPKKNNGDFSNIEIVPTNCFESHLYSAYKNDSSAYHLEKLDIDKKYAINSSFSD  130

P. berghei     124  FDLNIILIPSLYNIDKTIYCRCDNSKTKRELIKNDGENIKLQGKLGLVKIILNNQQNSPQ  183
                    F L I++IP+ Y   KTIYCRCDNSKT++ +    G++  L+GKLGLVKIIL NQ N
P. falciparum  131  FYLKILVIPNEYKSHKTIYCRCDNSKTEKNI---PGQDKILKGKLGLVKIILRNQYN---  184

P. berghei     184  NIYHITRSTQVGSLDNKV------IELKEGEIVHLKYDGKTRT-NFNCKEIINMKISIPL  236
                    NI  + ++  +  + NK       I+LKE +I+      +T   + NC+EI+N KI++
P. falciparum  185  NIIELEKTKPI--IHNKKDTYKYDIKLKESDILMFYMKEETIVESGNCEEILNTKINLLS  242

P. berghei     237  DYNLSMRMPTVFLKDINCKFHLIFSNVGGIANIVFKAKKTENIDGCDFTVPKGKGLYKNG  296
                    + N+ ++MP++F+ +INC       N      I  KA KT++IDGCDFT PKGKG+YKNG
P. falciparum  243  NNNVVIKMPSIFINNINCMLSSQDQN-NEKNYINLKADKTKHIDGCDFTKPKGKGIYKNG  301

P. berghei     297  FALSEINNDEE-ICTVHIGRGQNSNAAGLKCPYNLTPAHCFKHVLYEKKYKNGNNVFQTF  355
                    F +++I N+EE ICTVH+   +N   AG+KCPY L P +CFKHVLYEK+  +    ++TF
P. falciparum  302  FIINDIPNEEERICTVHLWNKKNQTIAGIKCPYKLIPPYCFKHVLYEKEI-DSQKTYKTF  360

P. berghei     356  LLDDVLRTVDIEYYYNAKLSAHIVGIPTIPEKSETVRCVCEHDGKKGIMELKISSSKNIF  415
                    LL DVL T +IEYY N K   +++ +PT PEK+  +RC+CE  GKK +MEL I+S+   +
P. falciparum  361  LLSDVLDTPNIEYYGNNKEGMYMLALPTKPEKTNKIRCICEQGGKKAVMELHIASTSTKY  420

P. berghei     416  ISFILLSIIVSIFYLF  431
                    IS  L+  ++ IFY++
P. falciparum  421  ISMFLIFFLIVIFYMY  436
```

Supplementary Figure 1. Phylogenic relationship and sequence analysis of P47 orthologs. (**A)** Neighbor-joining tree of P47 amino acid sequences from orthologs in different *Plasmodium* species; the sequences used in the alignment were (plasmodb ID): AK88_05126, C922_01956, PBANKA_1359700, PCHAS_1364300, PCOAH_00041200, PCYB_121690, PPRFG01_1348700, PGAL8A_00257900, PGSY75_1346800, PKNH_1254100, PRCDC_1345800, PRELSG_1251100, PVX_083240, PY04205, PmUG01_12018100, PocGH01_12016300, YYE_00614. (**B**) Protein blast showing the amino acid identity between Pfs47 and Pbs47.

A

> *P. berghei* Pbs47 Thioredoxin fusion protein sequence (T-Pbs47-FL)

MSDKIIHLTDDSFDTDVLKADGAILVDFWAEWCGPCKMIAPILDEIADEYQGKLTVAKLNIDQNPGTAPKYGIRGIPTLLLFKNGEVAAT
KVGALSKGQLKEFLDANLAGSGSGHSSGLVPRGSGMKETAAAKFERQHMDSPDLGTDDDDKAMADISDPNSSSVDKLAAAIFPNGYVCDF
KFNPLVNVLPSINTTGDIEEVGCTINNPSLSDYIALVCPKKNYNDYEHMEKVPSKCFSSNLYSPYKSEDSAHKLEELKIPEKYSISKDFS
DFDLNIILIPSLYNIDKTIYCRCDNSKTKRELIKNDGENIKLQGKLGLVKIILNNQQNSPQNIYHITRSTQVGSLDNKVIELKEGEIVHL
KYDGKTRTNFNCKEIINMKISIPLDYNLSMRMPTVFLKDINCKFHLIFSNVGGIANIVFKAKKTENIDGCDFTVPKGKGLYKNGFALSEI
NNDEEICTVHIGRGQNSNAAGLKCPYNLTPAHCFKHVLYEKKYKNGNNVFQTFLLDDVLRTVDIEYYYNAKLSAHIVGIPTIPEKSETVR
CVCEHDGKKGHHHHHH

> *P. berghei* Pbs47 Thioredoxin fusion *E. coli* codon optimized DNA sequence (T-Pbs47-FL)

ATGAGCGATAAAATTATTCACCTGACTGACGACAGTTTTGACACGGATGTACTCAAAGCGGACGGGGCGATCCTCGTCGATTTCTGGGCA
GAGTGGTGCGGTCCGTGCAAAATGATCGCCCCGATTCTGGATGAAATCGCTGACGAATATCAGGGCAAACTGACCGTTGCAAAACTGAAC
ATCGATCAAAACCCTGGCACTGCGCCGAAATATGGCATCCGTGGTATCCCGACTCTGCTGCTGTTCAAAAACGGTGAAGTGGCGGCAACC
AAAGTGGGTGCACTGTCTAAAGGTCAGTTGAAAGAGTTCCTCGACGCTAACCTGGCCGGTTCTGGTTCTGGCCATTCTTCTGGTCTGGTG
CCACGCGGTTCTGGTATGAAAGAAACCGCTGCTGCTAAATTCGAACGCCAGCACATGGACAGCCCAGATCTGGGTACCGACGACGACGAC
AAGGCCATGGCTGATATCTCGGATCCGAATTCGAGCTCCGTCGACAAGCTTGCGGCCGCAATATTCCCTAATGGATATGTCTGTGATTTT
AAATTCAACCCATTAGTTAATGTTCTTCCAAGTATAAACACAACTGGGGATATTGAAGAAGTAGGATGTACAATAAATAATCCTAGTTTA
TCAGATTATATTGCCTTGGTATGCCCCAAAAAAAATTATAACGACTATGAGCATATGGAAAAGGTACCGTCAAAATGTTTTTCGTCAAAT
TTATATTCTCCATATAAATCTGAAGACAGCGCACACAAATTAGAAGAACTTAAAATTCCAGAAAAATATTCAATATCAAAAGATTTTAGT
GATTTTGATTTAAATATTATATTAATTCCAAGTTTATATAATATTGATAAAACAATATATTGTAGGTGTGATAACTCCAAAACAAAACGC
GAGCTTATTAAAAATGATGGAGAAAATATAAAATTACAAGGAAAGTTAGGATTAGTCAAAATTATTCTAAACAATCAACAAAATTCTCCA
CAAAATATTTATCATATTACTCGTAGTACACAAGTAGGTTCACTTGATAATAAAGTTATTGAATTAAAAGAGGGAGAAATTGTTCATTTA
AAATATGATGGTAAAACCAGAACAAACTTTAATTGTAAGGAAATTATTAATATGAAAATATCTATTCCATTGGATTATAATTTATCTATG
AGAATGCCAACAGTATTTCTCAAAGATATTAATTGCAAATTCCATTTGATTTTTAGCAATGTTGGTGGCATTGCAAATATTGTATTTAAG
GCCAAGAAAACAGAAAACATAGATGGATGCGATTTTACAGTACCTAAAGGAAAAGGCTTATATAAAAATGGTTTTGCATTAAGTGAAATA
AATAATGATGAAGAAATATGCACTGTTCATATAGGTAGAGGTCAAAACAGTAATGCCGCAGGATTAAAATGCCCATACAATTTAACGCCT
GCTCATTGTTTTAAACATGTATTGTATGAAAAAAAATATAAAAACGGAAATAATGTATTTCAAACGTTTTTATTAGATGATGTTTTAAGA
ACTGTTGATATAGAATATTATTACAATGCCAAACTTTCTGCACACATTGTTGGTATACCAACTATACCTGAGAAATCCGAAACCGTACGA
TGTGTTTGTGAACATGATGGAAAAAAGGGACACCACCACCACCACCACTGA

B

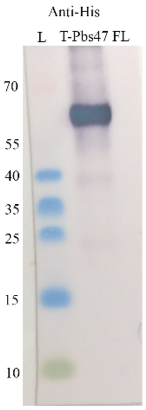

Supplementary Figure 2. Sequence, Expression and Purification of T-Pbs47-FL expressed in *E. coli* Shuffle T7. (**A)** Protein and DNA optimized sequence of T-Pbs47-FL. Thioredoxin fusion protein and His Tag are highlighted in grey. (**B)** Anti-His western blot of T-Pbs47-FL (thioredoxin fusion protein); lane 1, protein ladder (KDa); lane 2, purified soluble protein after in-column refolding and nickel affinity purification.

A

> *P. berghei* Pbs47 Domain 1 protein sequence **(Pbs47-D1)**

MIFPNGYVCDFKFNPLVNVLPSINTTGDIEEVGCTINNPSLSDYIALVCPKKNYNDYEHMEKVPSKCFSSNLYSPYKSEDSAHKLEELKI
PEKYSISKDFSDFDLNIILIPSLYNIDKTIYCRCDNSKTHHHHHH.

> *P. berghei* Pbs47 Domain 1 *E. coli* codon optimized DNA sequence **(Pbs47-D1)**

ATGATATTCCCTAATGGATATGTCTGTGATTTTAAATTCAACCCATTAGTTAATGTTCTTCCAAGTATAAACACAACTGGGGATATTGAA
GAAGTAGGATGTACAATAAATAATCCTAGTTTATCAGATTATATTGCCTTGGTATGCCCCAAAAAAAATTATAACGACTATGAGCATATG
GAAAAGGTACCGTCAAAATGTTTTTCGTCAAATTTATATTCTCCATATAAATCTGAAGACAGCGCACACAAATTAGAAGAACTTAAAATT
CCAGAAAAATATTCAATATCAAAAGATTTTAGTGATTTTGATTTAAATATTATATTAATTCCAAGTTTATATAATATTGATAAAACAATA
TATTGTAGGTGTGATAACTCCAAAACACACCACCACCACCACCACTGA

B

> *P. berghei* Pbs47 modified-Domain 2 protein sequence **(Pfs47-mD2)**

MKRELIKNDGENIKLQGKLGLVKIILNNQQNSPQNIYHITRSTQVGSLDNKVIELKEGEIVHLKYDGKTRTNFNAKEIINMKISIPLDYN
LSMRMPTVFLKDINAKFHLIHHHHHH.

> *P. berghei* Pbs47 modified-Domain 2 *E. coli* codon optimized DNA sequence **(Pbs47-mD2)**

ATGAAACGCGAGCTTATTAAAAATGATGGAGAAAATATAAAATTACAAGGAAAGTTAGGATTAGTCAAAATTATTCTAAACAATCAACAA
AATTCTCCACAAAATATTTATCATATTACTCGTAGTACACAAGTAGGTTCACTTGATAATAAAGTTATTGAATTAAAAGAGGGAGAAATT
GTTCATTTAAAATATGATGGTAAAACCAGAACAAACTTTAATTGTAAGGAAATTATTAATATGAAAATATCTATTCCATTGGATTATAAT
TTATCTATGAGAATGCCAACAGTATTTCTCAAAGATATTAATTGCAAATTCCATTTGATTCACCACCACCACCACCACTGA

C

> *P. berghei* Pbs47 Domain 3 protein sequence **(Pbs47-D3)**

MFSNVGGIANIVFKAKKTENIDGCDFTVPKGKGLYKNGFALSEINNDEEICTVHIGRGQNSNAAGLKCPYNLTPAHCFKHVLYEKKYKNG
NNVFQTFLLDDVLRTVDIEYYYNAKLSAHIVGIPTIPEKSETVRCVCEHDGKKGHHHHHH.

> *P. berghei* Pbs47 Domain 3 *E. coli* codon optimized DNA sequence **(T-Pfs47-D3)**

ATGTTTAGCAATGTTGGTGGCATTGCAAATATTGTATTTAAGGCCAAGAAAACAGAAAACATAGATGGATGCGATTTTACAGTACCTAAA
GGAAAAGGCTTATATAAAAATGGTTTTGCATTAAGTGAAATAAATAATGATGAAGAAATATGCACTGTTCATATAGGTAGAGGTCAAAAC
AGTAATGCCGCAGGATTAAAATGCCCATACAATTTAACGCCTGCTCATTGTTTTAAACATGTATTGTATGAAAAAAAATATAAAAACGGA
AATAATGTATTTCAAACGTTTTTATTAGATGATGTTTTAAGAACTGTTGATATAGAATATTATTACAATGCCAAACTTTCTGCACACATT
GTTGGTATACCAACTATACCTGAGAAATCCGAAACCGTACGATGTGTTTGTGAACATGATGGAAAAAAGGGACACCACCACCACCACCAC
TGA

D

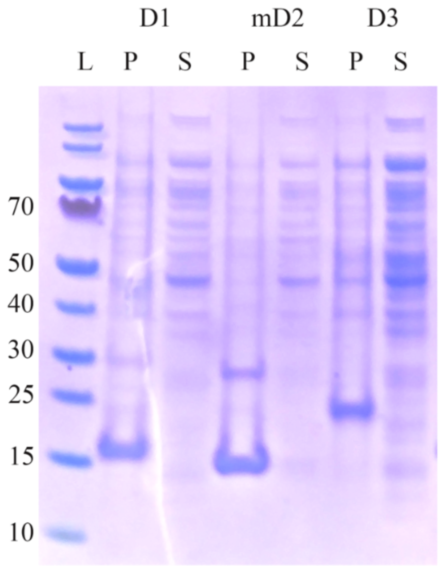

Supplementary Figure 3. Expression and purification of Pbs47-D1, Pbs47-mD2, and Pbs47-D3. Protein and DNA optimized sequences of (**A**) Pbs47-D1, (**B**) Pbs47-mD2, and (**C**) Pbs47-D3. His Tag are highlighted in grey. (**D**) Coomassie blue-stained SDS-PAGE gel illustrating the expressed proteins. Lane 1 corresponds to the molecular weight standards (KDa). Expression of individual domain was observed in the inclusion bodies pellet (P).

A

>*P. berghei* Pbs47 modified Domain 2 Deletion 1 protein sequence **(Del1)**

MKRELIKNDGENIKLQGKLGLVKIILNNQQNSPQNIYHITRSTQVGSLDNKVIELKEGEIVHLKYDGKTRTNFNAKEIINMKISIPLDYNLSM
RMPTVFLKDINAKFHLIHHHHHH

MILNNQQNSPQNIYHITRSTQVGSLDNKVIELKEGEIVHLKYDGKTRTNFNAKEIINMKISIPLDYNLSMRMPTVFLKDINAKFHLIHHHHHH

>*P. berghei* Pbs47 modified Domain 2 Deletion 1 *E. coli* codon optimized DNA sequence **(Del1)**

ATGATTCTGAACAACCAGCAAAACAGCCCGCAGAACATCTACCACATTACCCGTAGCACCCAAGTGGGCAGCCTGGACAACAAGGTTATCGAG
CTGAAAGAGGGTGAAATTGTGCACCTGAAGTACGATGGCAAAACCCGTACCAACTTCAACGCGAAGGAAATCATCAACATGAAGATCAGCATC
CCGCTGGACTATAACCTGAGCATGCGTATGCCGACCGTTTTCCTGAAGGATATCAACGCGAAATTTCACCTGATTCACCACCACCACCACTAA

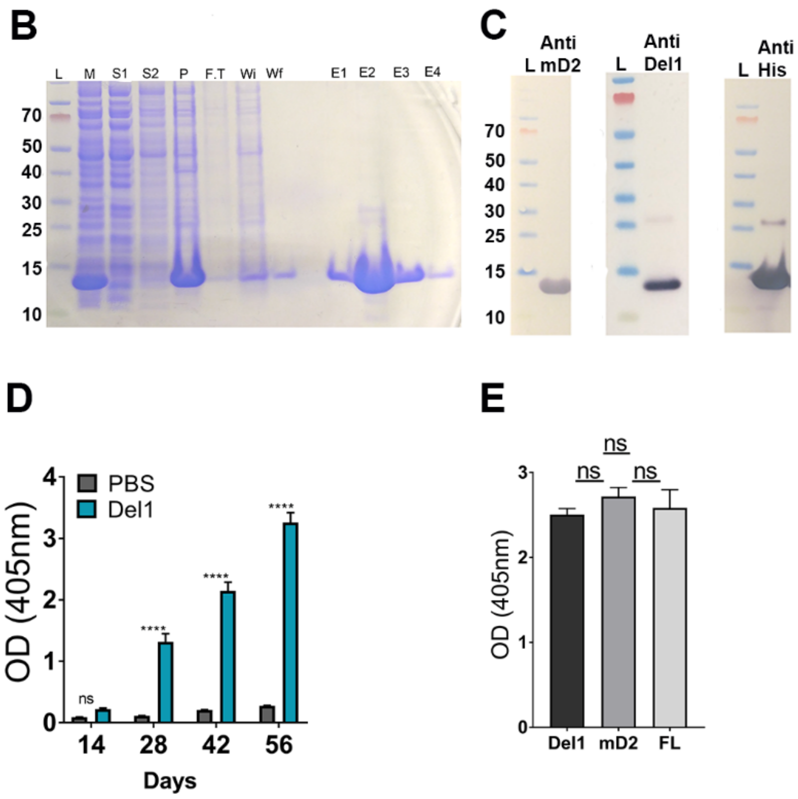

Supplementary Figure 4. (**A**) Schematic representation of the deleted region of Pbs47-mD2 (red) and the generated Del1. (**B**) Nickel affinity purification of Pbs47-Del1. M, protein ladder (kDa); L, cell lysate; S1 and S2 are supernatant after centrifugation; P, pellet; F, flow through; Wi, initial wash; Wf, final wash; E1, E2 and E3 are serial elution of Del1 protein. (**C**) Western blot showing the detection of Del1 by anti-mD2, anti-His, and anti-Del1 (**D**) ELISA from sera collected every two weeks shows that immunized mice produced stronger antibody responses throughout the immunization protocol (CTL= PBS immunized mice). (**E)** Anti-Del1 antibodies can detect both the full-length protein, T-Pbs47, and the modified domain 2, Pbs47-mD2. Sera was diluted 1:10000. Non-parametric t-test was used to compare between two groups: ns, non-significant; ****P < 0.0001.

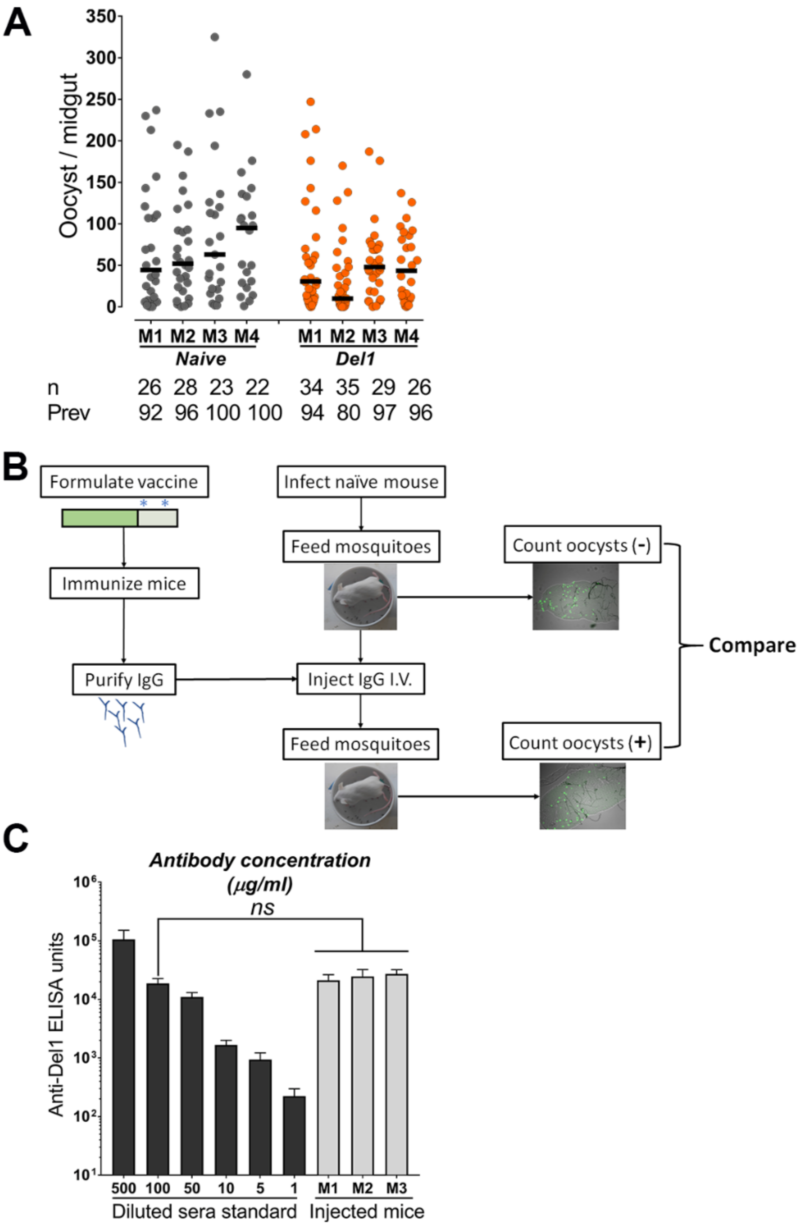

Supplementary Figure 5: **Passive immunization schematic and transmission-reducing activity of Del1.** **(A)** SMFA showing the variations of infections (oocyst/midgut) when using naïve and immunized mice-derived sera **(B)** Schematic of passive immunization protocol. Antibodies were purified from mice immunized with Pb-Del1 and stored at -80C. Naïve mice were infected with *P. berghei* parasites. After reaching the desired parasitemia, each mouse was fed to mosquitoes before and after antibody injection. Mean oocysts density was compared to indicate TRA induced by antibodies. **(C)** Determination of the biodistribution of IgG following passive immunization. ELISA showing the level of antibodies in a set of BALB/C mice following passive immunization with 5 µg IgG per gram mouse weight. The mice used in this experiment were of different age and weight to show consistency. Results shows replicated data of n=2 assays. Each dot represents the number of oocysts in individual mosquito and the lines indicate the medians per group. Number of mosquitoes dissected (n); Infection prevalence (Prev); Transmission-reducing activity (TRA) as percent inhibition of infection intensity relative to naïve control mice. This data represents the overall result from n=2 replicates.

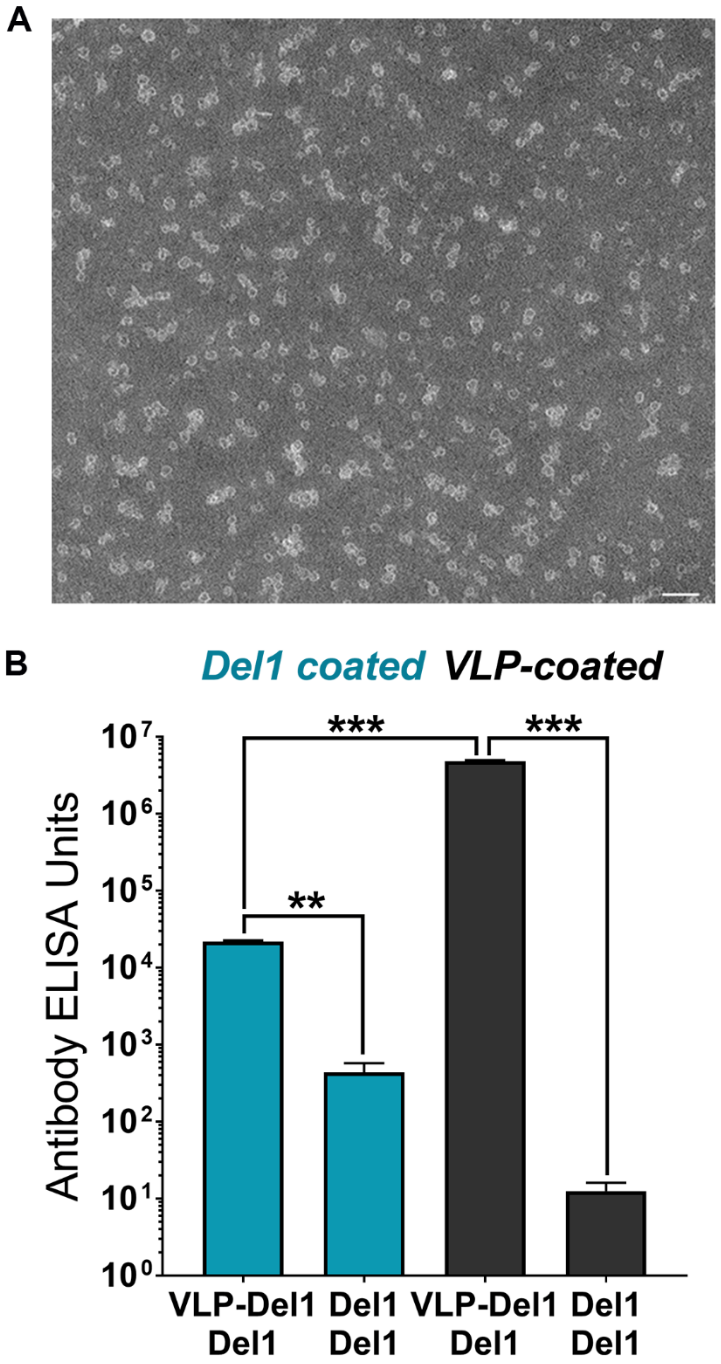

Supplementary Figure 6. Characterization and immunity of VLPs. (**A**) TEM of VLP-Del1 after negative staining with 2% uranyl acetate. Scale bar 100 nm (**B**) IgG ELISA reactivity of VLP-Del1/Del1 or Del1/Del1 measured using serial dilutions against Del1 and VLP. Errors bars represent mean absorbance value ± standard deviation of two biological replicate assays. Non-parametric t-test was used to compare between two groups: $^{**}P < 0.01$; $^{***}P < 0.001$; $^{****}P < 0.0001$.

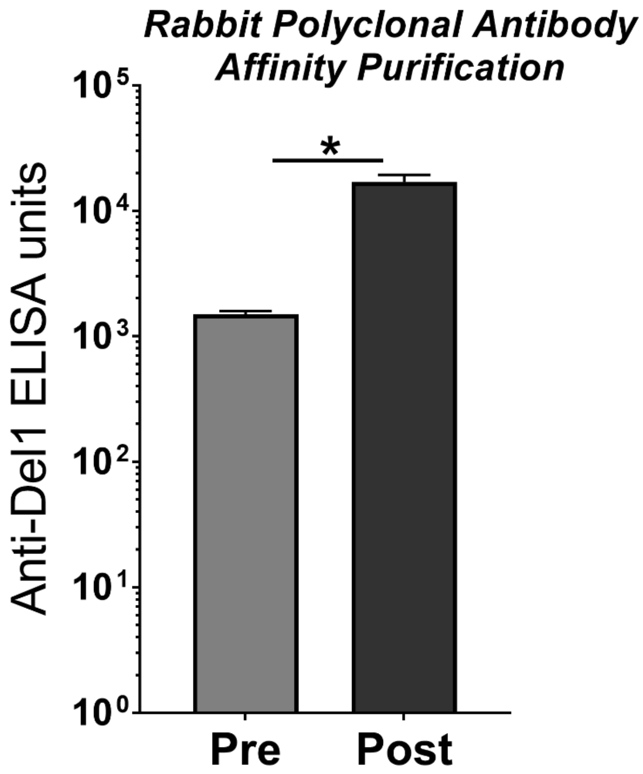

Supplementary Figure 7. ELISA showing the level of rabbit-derived Del1-specific antibody in polyclonal versus affinity purified antibody. Non-parametric t-test was used to compare between two groups: $^*P < 0.05$;
